# Workers with Occupational Contact Dermatitis: Work Outcomes and Return to Work Process in the First Six Months following Diagnosis

**DOI:** 10.1155/2011/170693

**Published:** 2011-05-08

**Authors:** D. Linn Holness

**Affiliations:** Department of Occupational Health and Keenan Research Centre, Li Ka Shing Knowledge Institute, St. Michael's Hospital and the Department of Medicine and Dalla Lana School of Public Health, University of Toronto, 30 Bond Street Toronto, ON, Canada M5B 1W8

## Abstract

Workers with occupational contact dermatitis may have poor outcomes that impact their health, work, and quality of life. While there is information available on overall return to work, little is known about the actual return to work process. The objectives of the study were to describe the return to work experience and work outcome in workers with contact dermatitis following diagnosis. 78 workers with occupational contact dermatitis were followed for 6 months after assessment. Information collected included clinical presentation and status, the return to work process and work outcomes. Six months after assessment, 38% were not working, almost all because of their skin problem. Of the 62% working 32% had changed job, most because of their skin problem. Limited advice to enable return to work and communication were reported. These findings suggest that there are gaps in return to work programs for occupational contact dermatitis and further research is needed.

## 1. Introduction

Workers with occupational contact dermatitis (OCD) may have adverse outcomes. Not only may they continue to have disease and their overall quality of life may be impacted, but they may also have significant work disruption. A number of studies have provided disease outcome information and more recently there have been studies demonstrating an impact on quality of life. There are some studies reporting on work outcomes in populations of workers with OCD and one study examined barriers to return to work [1–10]. Most studies examine work outcomes a number of years after diagnosis [1–8]. A recent study examined outcomes six months after diagnosis [9]. While overall work status has been reported, there is little information about the return to work process. The objective of the study was to describe the work outcomes and return to work process in workers with OCD over a six-month period following diagnosis.

## 2. Materials and Methods

The study was reviewed and approved by the Research Ethics Board of St. Michael's Hospital. Potential participants were informed of the purpose, activities, risks, and benefits of the study and their signed consent to participate was obtained. 

The individuals were recruited from the Occupational Health Clinic at St. Michael's Hospital. Individuals were referred to the clinic for diagnosis of OCD. Once the individual had been assessed and patch testing ordered, they were approached to participate in the study.

Patients were invited to participate if (a) they had a possible diagnosis of contact dermatitis, (b) were employed or had been employed but stopped work because of their skin disease, (c) were undergoing patch testing, and, d) had hand involvement. 100 subjects were recruited who met the inclusion criteria.

The workers were assessed at three points of time. The initial assessment took place when the worker was being patch-tested. This assessment collected information regarding the worker's clinical history and work status at the time of patch testing. Two follow-up assessments were done focusing on the immediate period following diagnosis. The first follow-up assessment was carried out at three months by telephone and included a brief questionnaire regarding work status. The second follow-up assessment was carried out at six months, in person or by telephone, and included a detailed questionnaire about the workplace and return to work, workers' compensation and health care utilization over the previous six months.

As the main purpose of the study was descriptive, the data were analyzed using standard statistical methods including means and frequencies.

## 3. Results

 One hundred workers were enrolled in the study and completed the initial assessment. As the workers were enrolled prior to final diagnosis, 78 were determined to have OCD following their assessment and patch testing. Of the 78 workers with a diagnosis of OCD, 75 completed the second assessment and 60 workers completed the third assessment. The results presented relate to the 78 workers with OCD.

### 3.1. Initial Assessment

The mean age was 40 with a range from 19 to 63 and 64% were male. The length of time with the rash prior to assessment at St Michael's was 25 months. All workers had hand involvement. The arm was affected in 26% and the face, neck, or leg in 12%. Almost all described itching, pain, redness, and scaling, while 86% noted cracking and bleeding and 74% blisters. When asked about the relationship between symptoms and work, 91% noted their skin was worse at work and 87% noted improvement on vacation. A past history of any atopic condition was reported by 56% of workers including 46% with allergies, 19% with hayfever, 15% with asthma, and 14% with eczema. Family history of atopic disease was also common with 53% reporting any atopic condition including 19% with eczema.

The mean total time at their current worksite was 79 months (range 2 to 238 months) and the mean time at their current job was 65 months (range 1 to 324 months). A variety of workplace exposures were reported. These included 91% with exposure to cleaning agents, 77% to metals, 72% to solvents, 46% to oils and greases, 37% to plastics, and 24% to other chemicals. Eighty-six percent reported wearing gloves at work and 21% reported wearing gloves while working at home.

A diagnosis of irritant contact dermatitis was made in 83% and of allergic contact dermatitis in 51%. Additional diagnoses included 8% with atopic dermatitis and 5% with psoriasis.

At the time of the initial assessment and patch testing 10% were not working. Of these 75% were not working because of their skin problem. Of those who were working, 83% were at the same job as when the problem started. If they were working but had changed job, 67% had done so because of their skin. Forty-four percent reported talking to someone at their workplace about a workers' compensation claim and 41% reported that a workers'compensation claim had been submitted.

### 3.2. Follow-Up Assessment at Three Months

At the three month follow-up interview we were able to contact 75 of the 78 workers. At this time 26% were not working, 95% of these because of their skin. Twenty-one percent reported that they had been terminated. Of those who were working, 78% were at the same job as when the problem started. If they were working but had changed job, all had changed jobs because of their skin. Information was obtained regarding advice received related to return to work (RTW) and the results are presented in Table 1. The minority of workers reported receiving recommendations regarding a change of job or job modification and, even if advice had been provided, it was not always implemented.

Information was obtained about communication during the RTW process (Table 1). Communication between the various parties involved in RTW was reported by a minority of workers. Workers reported that the physician wrote a letter or talked to the employer for 23% of the workers. Forty-four percent of the workers talked with their supervisor; if the worker belonged to a union, 31% talked with their union; 19% were involved in a meeting concerning their RTW involving their supervisor and others; 16% had some discussion about potential problems with RTW; and for 4% there was some discussion of their RTW with their coworkers.

### 3.3. Follow-Up Assessment at Six Months

At the six month follow-up interview we were able to contact 60 of the 78 workers. To provide comparability of results across the 3 visits, the results of work status at the initial assessment, at three months and six months for the 60 workers that were assessed at all three visits are presented in Table 2. At six months 38% were not working, 96% of these because of their skin. 15% were receiving workers' compensation benefits, 3% employment insurance benefits, and for 10% the company had changed their job and no compensation claim had been filed. Of those who were working, 68% were at the same job as when the problem started. If they were working but had changed job, 92% had done so because of their skin. With respect to workers' compensation, 69% reported submitting a claim. Of those who submitted a workers'compensation claim, 70% were accepted, 6% denied, 15% pending, and 9% did not know the status of their claim. 58% had talked to their adjudicator.

In addition to work status, information regarding work practices, communication amongst the parties involved in RTW and interactions with health care providers were obtained. These results are presented in Table 3. A lack of communication with key parties continued to be reported. Follow-up with workplace health care providers was also limited. 

## 4. Discussion

Previous studies have demonstrated significant work disruption in workers with OCD [1–9]. The results from this study confirm the impact on work. Some workers had already experienced work disruption by the time of their initial assessment and significant further change in work status occurred in the six months following diagnosis. At the time of initial assessment, 10% were off work, most because of their skin. By three months, the percentage not working had increased to 26% and at 6 months, the percentage not working had increased to 38%.

At three months after diagnosis, the job modifications suggested including the use of gloves, changing the type of glove used and changes in skin care were suggested in the minority of cases and implemented in even fewer. When communication between the various parties was examined at three months, 44% had talked with their supervisor and this increased to 69% by six months. Similarly, at three months 31% of union members had talked with their union and this increased to 42% at 6 months. Return to work was rarely discussed with co-workers. These findings all suggest that there are significant gaps in the RTW process. These gaps include lack of advice provided regarding needed job or workplace change, lack of implementation of modification and suboptimal between the workplace parties who are involved in the RTW process. There is also limited interaction with health care providers in the workplace. Follow-up visits with their family physicians and, dermatologists, reported elsewhere, were 62% and 39%, respectively, even though workers are advised to see their physicians for follow-up [11]. These findings suggest that there is significant room to improve the RTW (or stay at work) process. Further research, focused on the various aspects of RTW, including the identification of barriers and facilitators are needed to improve the RTW process and ultimately achieve better work outcomes for workers with OCD.

## Figures and Tables

**Table 1:** Return to work advice and return to work process reported at 3 month follow-up.

(*N* = 75)	
	% reporting
Work modification	

Avoid particular exposure	
Suggested	12%
Implemented	8%
Use different personal protective equipment	
Suggested	12%
Implemented	8%
Start using gloves	
Suggested	20%
Implemented	19%
Change types of gloves used	
Suggested	16%
Implemented	12%
Change skin care regimen	
Suggested	11%
Implemented	11%

Return to work communication	

Physician wrote letter or talked with employer	23%
Worker talked with supervisor about return to work	44%
Talked with union about return to work (if belonged to union)	31%
Met with supervisor to discuss return to work	19%
Discussed potential problems related to return to work with anyone	16%
Discussed return to work with co-workers	4%

**Table 2:** Work status at Each visit for 60 workers assessed at all 3 visits.

Percent reporting			
Work status	Intial visit	3 Months	6 Months
Not working	12%	29%	38%
%not working because of skin	71%	100%	96%
Working	88%	71%	62%
Working but changed jobs	78%	74%	67%
%changed job because of skin	64%	100%	92%

**Table 3:** Return to work status, work practices and return to work process reported at 6 month follow-up.

(*N* = 60)	
	% reporting
Work status	

Working	62%
% working with same employer	89%
% working with same employer, same job	73%

Work modifcation (Same employer, same job)	

Job modifications suggested and impelmented	74%
Job modifications suggested but not implemented	13%

Work practices	

Use gloves	81%
Use gloves all the time	11%
Changed skin care regimen	32%
Use emollients	68%
At work	57%
At home	68%

Return to work communication	

Worker talked with supervisor about return to work	69%
Talked with union about return to work (if belonged to union)	42%

Workplace health care utilization	

Saw workplace nurse about skin problem	11%
If saw workplace nurse previously about skin problem, saw again	38%
Saw workplace doctor about skin problem	6%
If saw workplace doctor previously about skin problem, saw again	13%
